# Instrumental variable meta-analysis of individual patient data: application to adjust for treatment non-compliance

**DOI:** 10.1186/1471-2288-11-55

**Published:** 2011-04-21

**Authors:** Branko Miladinovic, Ambuj Kumar, Iztok Hozo, Benjamin Djulbegovic

**Affiliations:** 1 Center for Evidence Based Medicine and Health Outcomes Research, University of South Florida, Tampa, FL, USA; 2 H Lee Moffitt Cancer Center & Research Institute, Tampa, FL, USA; 3 Department of Mathematics, Indiana University Northwest, Gary, IN, USA

## Abstract

**Background:**

Intention-to-treat (ITT) is the standard data analysis method which includes all patients regardless of receiving treatment. Although the aim of ITT analysis is to prevent bias due to prognostic dissimilarity, it is also a counter-intuitive type of analysis as it counts patients who did not receive treatment, and may lead to "bias toward the null." As treated (AT) method analyzes patients according to the treatment actually received rather than intended, but is affected by the selection bias. Both ITT and AT analyses can produce biased estimates of treatment effect, so instrumental variable (IV) analysis has been proposed as a technique to control for bias when using AT data. Our objective is to correct for bias in non-experimental data from previously published individual patient data meta-analysis by applying IV methods

**Methods:**

Center prescribing preference was used as an IV to assess the effects of methotrexate (MTX) in preventing debilitating complications of chronic graft-versus-host-disease (cGVHD) in patients who received peripheral blood stem cell (PBSCT) or bone marrow transplant (BMT) in nine randomized controlled trials (1107 patients). IV methods are applied using 2-stage logistic, 2-stage probit and generalized method of moments models.

**Results:**

ITT analysis showed a statistically significant detrimental effect with the use of day 11 MTX, resulting in cGVHD odds ratio (OR) of 1.34 (95% CI 1.02-1.76). AT results showed no difference in the odds of cGVHD with the use of MTX [OR 1.31 (95%CI 0.99-1.73)]. IV analysis further corrected the results toward no difference in the odds of cGVHD between PBSCT vs. BMT, allowing for a possibility of beneficial effects of MTX in preventing cGVHD in PBSCT recipients (OR 1.14; 95%CI 0.83-1.56).

**Conclusion:**

All instrumental variable models produce similar results. IV estimates correct for bias and do not exclude the possibility that MTX may be beneficial, contradicting the ITT analysis.

## Background

Intention-to-treat (ITT), per protocol (PP), and as treated (AT) methods have commonly been used to analyze data from experimental studies involving human subjects. ITT analysis includes all patients regardless of whether they adhered to the prescribed protocol and is recommended as the least biased method to estimate treatment effects in randomized controlled trials (RCTs)[1-4]. Excluding patients from the analysis who do not adhere to the assigned treatment is called per protocol (PP) analysis. It is designed to measure the treatment effects only in patients who complied with the treatment and ignores the ones who were intended to receive treatment but did not actually receive it [5-7]. Not discarding information and analyzing patients according to the treatment received rather than intended is called as treated (AT) or treatment received analysis [4,7]. On its face value PP and AT analysis seem to be reasonable alternatives to ITT. However, both estimates can be unreliable because non-compliance to the protocol cannot be assumed random and may be related to many factors, which may include adverse events, prognosis, etc. and lead to selection bias compromising the purpose of randomization.

Differences in the calculated estimates using ITT, PP and AT methods can be considerable[6]. A recent study comparing treatment effects using ITT versus PP methods concluded that on average, the PP estimate (log odds ratio [OR]) is 1.25 times the ITT estimate [8]. The choice then seems to be between ITT analyses that eliminate selection bias and produces conservative estimates in favor of no treatment effects versus PP analyses that aim to produce actual but biased treatment effects.

As an alternative to ITT, PP or AT analysis, instrumental variable (IV) methods have been proposed [6,9]. IV analysis derives potentially unbiased estimates of treatment effects and has been extensively discussed and applied in the medical literature, both in the context of individual RCTs [10-12] and observational studies [13-17]. However, the IV methodology based on the treating center prescribing preference (CPP) has not been applied in the context of individual patient data meta-analyses (IPD MA), which has been described as the gold standard for combining evidence from existing clinical trials [18-20]. Specifically, the effects of unaccounted confounding variables in the context of RCTs (e.g. effect of co-interventions in one arm versus other) have not been systematically evaluated. We are interested in applying the IV methodology in the context of IPD MA and AT data. Specifically, our objective is to test the strength of CPP as an instrument and obtain less biased estimates of the effect of methotrexate (MTX) on chronic graft-versus-host-disease (cGVHD) in transplant patients with hematological malignancies.

## Methods

Previously collected IPD from nine separate randomized controlled trials were used to address the objective of this study [21]. The study was approved by the University of South Florida Institutional Review Board, which is accredited by the Association for the Accreditation of Human Research Protection Programs. Transplant patients were randomized to receive either peripheral blood stem cell transplant (PBSCT) or bone marrow transplant (BMT). Chronic GVHD is one of the most serious complications of stem cell transplantation that is associated with significant morbidity and mortality. The incidence of cGVHD is significantly increased in the patients who receive PBSCT [22], which on the other hand may provide benefits in terms of the increases in the overall and disease free survival. In order to counter the negative effects of cGVHD, particularly related to the use of PBSCT, some centers preventatively administered four doses of MTX (on days 1, 3, 7, and 11), while others administered three doses (on days 1, 3, and 7). It is not clear if the fourth dose of MTX provides an additional prophylactic effect on the incidence of cGVHD. Three centers prescribed three doses of MTX and six centers prescribed four doses of MTX to all their patients. Out of 1107 patients, 135 had missing values for cGVHD and were therefore not evaluable for the outcome and excluded. The analysis was not adjusted for the missing values of cGVHD, as attempts to impute data would produce results using assumptions that currently cannot be justified on a theoretical or empirical basis. We re-analyzed the data using IV methodology and center prescribing preference (CPP) as the instrument to correct for non-compliance. The distribution of CPP and cGVHD by the number of doses of MTX actually received is summarized in Table 1.

**Table 1:** CPP and observed cGVHD versus MTX received for 972 evaluable patients

CPP	MTX	cGVHD	Total
Four doses	Four doses	275	441
	
	Three doses	55	84

Three doses	Four doses	0	0
	
	Three doses	264	447

Preference based instruments have been used in literature, but never in the context of IPD MA (for a discussion on preference based instruments see [23-25]). The application of IV analysis rests on the idea that given treatment (MTX), outcome (cGVHD), and a set of measured and unmeasured confounders, there exists a variable such as CPP, which is related to the treatment but not to the outcome, except indirectly through the treatment. We used CPP as the instrument keeping in mind that CPP sufficiently varies among centers that prescribe treatment. Although the allocation of MTX was not randomized, the natural variation in CPP, creates a "pseudo-randomizing" process by which patients got assigned to different treatment groups. This argument is identical to the one utilized in the context of studies of adverse drug effects (ADE) where physicians who prescribed the drug could not predict ADE and make a choice on the basis of risk factors; however, the difference in adverse effects could be ascribed with confidence to the drug [26]. In this sense, prescribing preference at the center or physician level can indeed be thought of as a natural randomizing instrument [27].

**Figure 1:**
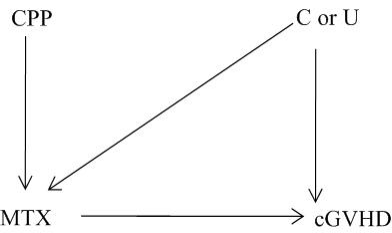
**Instrumental variable causal diagram**.

Figure 1 shows a causal diagram that describes the following three conditions that CPP must meet in order to qualify as an instrument:

1) Instrument (CPP) is independent of any measured confounder C or unmeasured confounder U, between treatment (MTX) and outcome (cGVHD)

2) CPP is associated with treatment MTX

3) CPP is independent of cGVHD given MTX and confounders (measured C and unmeasured U)

The first condition is the most difficult to justify in practice. In the case of our observational data, the argument is that CPP exhibits natural variation across different centers and introduces a natural randomizing effect. The second and third conditions are easier to justify, because the assignment of MTX is related to the centers' preference apriori and CPP affects cGVHD only through the centers' influence on the administration of MTX. This also satisfies another criterion-referred to in literature as *monotonicity *[28]-that no trial center would assign the opposite dose than what the protocol called for. In the context of our study, the violation of this criterion is highly unlikely.

Conditions 1-3 are satisfied in a randomized controlled trial, if equal treatment assignment becomes the instrument, whereby MTX would become treatment received[10]. In this case, the treatment assignment would affect the treatment received, but would not fully determine it, as some patients will inevitably not receive treatment due to voluntary refusal, non-compliance, treatment switch, or administrative error. Since MTX was not randomly assigned, in this non-experimental setting causal inference relies on the assumption that no unmeasured confounders exist, which as noted already may be difficult to control for in practice [29]. As randomization in clinical trials allows for valid inference in the presence of unmeasured covariates, under regression models without misspecification IV analysis provides an unbiased estimate in the presence of unmeasured covariates and potential confounders [30,31]. This does not hold for nonparametric analysis.

Two approaches have commonly been used in IPD MA: the two-stage approach in which treatment effects are analyzed within a trial and then pooled across all available trials, and the one-stage approach, where all the trials are combined and the pooled estimate is calculated stratified by trial [32,33]. The two methods for conducting IPD MA produce similar results, though the one-stage approach is rarely used[34-38]. Since the IV methodology we applied is based on the one-stage approach, we assessed the reproducibility and the equivalence of the approaches using our previously reported results [39,40]. Using the two-stage methodology, previous results reported that the overall survival was significantly better among recipients of PBSCT compared to BMT in studies where four doses were prescribed (OR = 0.67, 95% CI 0.52-0.88, P = 0.004). There was no difference in survival where only three doses of MTX were prescribed (OR = 1.19, 95% CI 0.89-1.60) [21]. Using 1-stage regression methodology we calculated odds ratios to be equal to 0.61 (0.51-0.72) and 1.26 (0.98 - 1.60) in studies that used four versus three doses of MTX, respectively.

We chose to use the one-stage approach for computational reasons and because the IV methodology applied to non-experimental data in the context of structural equation modeling allows for simultaneous causal modeling of MTX received as the outcome for MTX assigned and predictor of cGVHD occurred. If we let X be treatment (MTX), Y be outcome (cGVHD), C be one or more measured confounders (Trial and BMT vs. PBSCT allocation) and Z be the instrument (CPP), then for coefficients α_i _and β_i_, and errors ε_i_, the following equations are solved simultaneously:

In step one, the predictor variable was regressed on the instrument CPP and measured confounders (trial and allocation to PBSCT and BMT), and in step two the outcome was regressed on the instrumented predictor (MTX) and measured confounders. In our study both MTX and cGVHD are dichotomous variables.

Even though prescribing preference has been shown to be a strong instrument in past studies [15,41], we tested the strength of CPP as an instrument statistically using the partial F test statistic and Shea partial correlation coefficient r^2 ^[42]. The partial F statistic has the null hypothesis that the coefficient for the instrument effect in the first-stage regression is zero. The Shea partial correlation coefficient is the square of the partial correlation between the instrument and the treatment, conditional on other covariates in the model. The partial F statistic greater than 10 and a reasonable value of r^2 ^indicate that the instrument is not weak and contributes substantially to the prediction of treatment [15,43].

Three classes of two-step regression models have been proposed to implement IV analysis in the context of regression modeling of dichotomous outcomes such as ours (MTX and cGVHD), where odds ratios are of interest: two stage logistic equation, Probit and generalized method of moments (GMM) [17,44]. In two-step logistic IV modeling, the first logistic equation predicts the effects of instrument(s) and confounder(s) on the dichotomous treatment, whereas the second logistic equation models the dichotomous outcome in terms of the treatment and confounders.

Probit models are also two-step, but as opposed to two-step least squares they model probabilities directly and are restricted on [0,1], so that the system of Probit equations can be expressed as:

where I(.) is the indicator function which returns 0 if the condition is not met and 1 if it is. Since two-step least squares modeling may not return values in the 0-1 range, the Probit model has been suggested as the best alternative to modeling dichotomous data and has been preferred in the economics literature [17]. All models have been shown to provide similar estimates in past studies [16,17]. The coefficients of Probit models are not interpretable as logarithms of odds ratios (as is the case with logistic regression), but it has been shown that multiplying probit coefficients by 1.6 or 1.8 we get approximate logistic coefficients [45].

Lastly, GMM model estimates were derived by making assumptions about the moments of the error term under the mean logistic regression model

where

In particular, given outcome Y (cGVHD), treatment X (MTX) and instrument Z (CPP), the generalized method of moments (GMM) model estimates parameters by assuming the following:

i) The residuals should sum to zero:

ii) The errors ε must be uncorrelated with the confounders C:

iii) The errors ε must be uncorrelated with the instrument Z:

The GMM methods rely on the estimation of moments and are robust in that they do not make distributional assumptions of maximum likelihood. The parameters are estimated using the Newton-Raphson iterative methods. The standard errors of the two-step logistic and two-step Probit models cannot be expressed in closed form and were calculated using bootstrapping methods (using 1000 iterations).

All the analyses were done using STATA statistical software and ivreg2 module [46,47].

## Results

**Figure 2:**
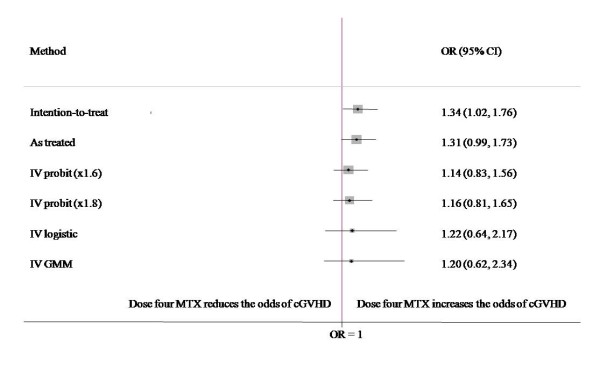
**Summary forest plot of estimated ORs, demonstrating the correcting effect of CPP as an instrument**.

Assessment of the strength of CPP as an instrument resulted in the partial F statistic of 18.10 and Shea partial r^2 ^of 0.69 suggesting choice of CPP as a strong instrument. Results of the study by Stem Cell Trialists' Group reported a significant increase in the odds of developing cGVHD in patients treated with PBSCT, irrespective of whether patients received three or four doses of MTX. Therefore, treatment allocation to PBSCT versus BMT was included in all three models as a confounding control covariate and to preserve the effects of the original randomization. A forest plot summarizes the distribution of OR estimates (Figure 2). According to the ITT method, the OR for all the patients, regardless of whether they received dose four MTX or not, was 1.34 (95% CI 1.02-1.76). It is important to note that the outcome cGVHD is a "bad" event. The ITT analysis counted those who did not receive the fourth dose of MTX as if they actually did, thus making it appear as if giving the fourth dose was increasing the odds of developing cGVHD. This is similar to what happens in non-inferiority trials where the ITT may bias estimates away from the null. The OR using AT analysis was 1.31 (95% CI 0.99-1.73). IV OR estimates range from 1.14 (95% CI 0.83-1.56) to 1.22 (95% CI 0.64-2.17) and suggest that the odds may be reduced by as much as 20%.

## Discussion

To our knowledge, this is the first paper that assesses the use of IV analysis in the context of IPD MA. We show how IV methods may be applied to correct for bias in observational data in IPD MA. We also show that center prescribing preference is a strong instrument. ITT analysis suggests that the fourth dose of MTX is detrimental and that physicians should seemingly not administer it. Per protocol, as well as IV estimates, support the conclusions of a previous study that reported no treatment difference with use of the fourth dose of MTX. However, the IV estimates based on center prescribing preference show a substantial decrease in odds of cGVHD compared with both ITT and AT estimates. In fact, IV IPD MA further corrected the results toward no significant difference in the odds of cGVHD adjusted for PBSCT vs. BMT groups, suggesting no effect of the fourth dose of MTX in preventing cGVHD in PBSCT recipients[22]. However, our goal here is not to develop practice guidelines for the use of MTX in the prevention of cGVHD. Our main objective is to show that IV analysis may offer an alternative to ITT analysis and that IV analysis is doable in a meta-analytic setting, which has not previously been done. We are also aware that based on IV analysis practicing physicians would obtain a different advice than based on ITT analysis.

Our study has some limitations. For example, we have not addressed the complexities of IV analysis that may involve multiple instruments, multiple regressors, or effects of other measured confounders. The objective was limited to the bias correction in AT data using only center prescribing preference as the instrument. Also, the two-step regression modeling we used is less efficient that the standard adjustment methods used and will generally produce wider confidence intervals, as Figure 2 clearly shows.

The key difficulty in conducting an IV analysis is finding and justifying a strong instrument, especially if the research questions revolve around multiple instruments and predictors. The assumptions of the existence of high level of correlation between the IV and the exposure, and zero correlation between the IV and the outcome must be justified. This is especially difficult for the latter as it is often impossible to do so on empirical grounds [27]. These issues need further exploration in the context of IPD MA.

## Conclusion

Our findings demonstrate that IV analysis can be applied to IPD MA of randomized and observational data. We recommend that IV methods for confounding control should be considered when conducting a meta-analysis of randomized controlled trials or observational studies, regardless of whether the analysis is based on aggregate or individual patient data.

## Competing interests

The authors declare that they have no competing interests.

## Authors' contributions

BM applied the statistical analysis, drafted the manuscript and participated in the discussions. AK participated in the discussions and helped write the manuscript. IH helped with the statistical analysis and interpretation of the results. BD conceived of the problem and participated in its framing and interpretation, and helped BM draft the manuscript. All authors read and approved the final version of the manuscript.

## Pre-publication history

The pre-publication history for this paper can be accessed here:

http://www.biomedcentral.com/1471-2288/11/55/prepub
